# A CRISPR-HITI strategy approach to improve CHO cell viability by modifying the 3'UTR of Caspase 8 Associated Protein 2

**DOI:** 10.22099/mbrc.2024.50513.2000

**Published:** 2025

**Authors:** Soofia Sorourian, Abbas Behzad-Behbahani, Mohsen Forouzanfar, Mojtaba Jafarinia, Fatemeh Safari

**Affiliations:** 1 Department of Biology, Marvdasht Branch, Islamic Azad University, Marvdasht, Iran; 2 Diagnostic Laboratory Sciences and Technology Research Center, School of Paramedical Sciences, Shiraz University of Medical Sciences, Shiraz, Iran

**Keywords:** CASP8AP2, CHO Cells, Gene Editing, Homologous Recombination, Apoptosis

## Abstract

Chinese Hamster Ovary (CHO) cells are essential in biopharmaceutical manufacturing. Scientists use CRISPR to enhance productivity. mRNAs contain UTRs that regulate gene expression, affecting protein abundance. Targeting these regions creates desirable knockout cells. The Caspase 8 Associated Protein 2 (*CASP8AP2*) gene is a promising target for improving host cell viability. This study used the CRISPR-Homology-Independent Targeted Integration (HITI) strategy to modify the 3′UTR region of the *CASP8AP2* gene in CHO cells. The aim was to evaluate the effects of *CASP8AP2* silencing on cell proliferation, viability, apoptosis, and the cell cycle. *CASP8AP2* silencing was assessed post-modification by extracting genomic DNA from modified and unmodified CHO cells, followed by PCR and sequencing to confirm deletions. Cell proliferation and viability were measured using MTT assays, and cell cycle analysis was performed via flow cytometry. Apoptosis was evaluated through Annexin V/PE staining and flow cytometry, with apoptosis resistance assessed by determining the IC_50_ of sodium butyrate. Results showed *CASP8AP2* deletion did not affect cell proliferation or the cell cycle but improved CHO cell viability and increased resistance to apoptosis. The IC_50_ for sodium butyrate was higher in *CASP8AP2* knockout cells (7.84 mM) compared to native cells (3.43 mM), indicating enhanced apoptosis resistance. This study highlights *CASP8AP2*'s role in apoptosis regulation without impacting cell proliferation or the cell cycle. *CASP8AP2* deletion enhances viability and resistance to apoptosis, suggesting it as a target for improving recombinant protein production. Further research is needed to elucidate the molecular mechanisms and develop therapeutic strategies based on this approach.

## Introduction

Chinese Hamster Ovary (CHO) cells have become the workhorse of the biopharmaceutical industry, thanks to their high protein expression levels. These cells are particularly well-suited for the production of monoclonal antibodies, which are used in a wide range of therapeutic applications. Researchers are constantly seeking ways to enhance the productivity of CHO cells, and one of the most promising strategies involves the use of genome editing techniques. Among these, the CRISPR-Cas9 system has emerged as a powerful tool for precise and efficient genetic manipulation [1]. A key step in modifying the genome of CHO cells using CRISPR-Cas9 is the identification of suitable target locations [2]. Guide RNAs (gRNAs), which direct the Cas9 enzyme to the desired genomic location, often target the first coding exons of genes. This strategy is particularly effective for protein-coding genes. However, there can be limitations in gRNA design that may necessitate targeting other regions of the genome [3]. 

The 3’ untranslated region (3’UTR) of genes is known to contain regulatory elements that control various aspects of protein biology, including its abundance, localization, and function. The region extending from the stop codon to the poly(A) tail includes sequences that are essential for mRNA processing [4]. The poly(A) tail plays a crucial role in regulating mRNA stability, translation, and degradation, thereby serving as a key mechanism for controlling gene expression [5]. Given this, targeting the poly(A) signal sequence could be a powerful strategy for gene deletion, especially when there are limitations in directly targeting the coding sequences of a gene (Fig. S1).

The caspase 8 associated protein 2 gene (*CASP8AP2*) has been identified as an effective target for increasing recombinant protein expression using small interfering RNAs (siRNAs). By targeting the *CASP8AP2* gene in the 3’UTR of CHO cells, it may be possible to disrupt or modify the poly(A) signal. This could potentially affect mRNA stability, translation efficiency, and ultimately, protein production levels [6]. 

*CASP8AP2*, a protein of high molecular weight, participates in numerous cellular activities. It triggers the extrinsic pathway of apoptosis and, as a multifunctional protein, it also contributes to necrosis instigated by tumor necrosis factor-alpha (TNF-α) [7]. It also participates in the processing and regulation of histone-encoding mRNAs, contributes to cell cycle regulation, and activates important transcriptional regulators [8]. 

Homology-independent targeted integration (HITI) is a cutting-edge CRISPR-based gene insertion technique. The HITI system allows for specific gene targeting with simultaneous knockout/knock-in [9]. The HITI system has an antibiotic selection marker and/or fluorescence, thus allowing the user to select target cells based on the fluorescent marker GFP [10]. HITI technology is used for precise genetic modification and insertion of large gene sequences which facilitates the selection of knockout clones and genetic alterations [9]. However, integrating CRISPR-Cas9 with HITI poses challenges such as unwanted genomic changes, mutations, or genomic instability [11]. To alleviate these concerns, researchers could design multiple gRNAs simultaneously. 

The major goal of this research was to confirm the deletion of the *CASP8AP2* gene and produce cells with increased cell viability and resistance to apoptosis. To achieve this, the *CASP8AP2* protein in CHO-K1 cells was knockout using The CRISPR/Cas9 genome editing tool, combined with the HITI approach.

## MATERIALS AND METHODS

**Designing of gRNAs and cloning in CRISPR and donor vectors: **A pair of gRNAs was utilized to disrupt the entire 3'UTR. The upstream gRNAs were strategically positioned near the termination codon to ensure precise cleavage in two sites. The Cas9 cleavage site is anticipated to be situated between nucleotide three and four upstream of the protospacer adjacent motifs (PAMs) site [12]. For implementing the CRISPR-HITI strategy, a set of normal gRNAs and their PAM+ gRNAs were used to design the CHOPCHOP online tool. gRNAs with the lowest number of off-targets, usually with one or two mismatches to potential target sites, were selected for CRISPR/Cas9-mediated gene editing specificity. This approach minimizes the chances of off-target mutations and translocations, which can lead to unintended consequences in the genome [12]. 

This study used two sgRNAs named gRNA-1 and gRNA-2 whose sequences are shown in Table 1. These gRNAs were directed towards the *CASP8AP2* gene's 3'UTR area (Genbank accession number: NW_003613592) in CHO cells. This study used the pSpcas9 (BB) 2A-Puro (PX459) V2.0 vector (Addgene, Plasmid #62988) as the main plasmid, into which the standard gRNAs were cloned, with the PX459 vector obtained from the Royan Research Institute located in Tehran (Iran). The PX460-1 vector was utilized for cloning the PAM+gRNAs that included an enhanced GFP expression cassette driven by a CAG promoter along with a polyadenylation signal, as well as a U6 promoter-based gRNA insertion site. The integration of the sgRNAs into the vector was subsequently confirmed by colony PCR utilizing certain primers. The cloning process followed a methodology outlined in a protocol by Ran et al [13]. PCR analysis was used to verify that the recombinant plasmids utilized in this study, as presented in Table 1 and Table S1 contained the intended gRNA sequences.

**Table 1:** List of sgRNAs and their corresponding sequences with PAM

**Name**	**Sequences**	**Annealing Temperature (°C)**
gRNA-1	Fw: 5′CACCGGACTAGATGATTATCGTGCT3′ Rev: 5′ AAACAGCACGATAATCATCTAGTCC3′	**66** **70**
gRNA-1 + PAM	Fw: 5′CACCGGACTAGATGATTATCGTGCTTGG3′ Rev: 5′AAACCCAAGCACGATAATCATCTAGTCC3′	**63** **67**
gRNA-2	Fw: 5′CACCGGATTCCTCATGTTTTAAATG3′ Rev: 5′AAACCATTTAAAACATGAGGAATCC3′	**63** **67**
gRNA-2 + PAM	Fw: 5′CACCGGATTCCTCATGTTTTAAATGAGG3′ Rev: 5′AAACCCTCATTTAAAACATGAGGAATCC3′	**59** **64**

**Culture of CHO cell: **This study employed the CHO-K1 cell line, which was obtained from Tehran, The Iranian National Center for Genetic and Biologic Resources. For maintenance and culture, the cells were cultured in RPMI-1640 medium (Gibco, USA), 10% FBS (Atlanta Biologicals, USA) added as a supplementary component and 1% streptomycin (Gibco, USA) as inhibit microbial contamination. The cultures were incubated at 37°C with a standard humidity condition of 5% CO2.

**Cellular transfection: **CHO-K1 cells at passage three were plated in 24-well culture plates, with each well receiving 5 × 10^4^ cells. After 24 hours of incubation, the cells reached an approximate confluence of 80%. Subsequently, Lipofectamine® 3000 (Thermo Fisher Scientific, USA) were used for transfection of four vectors containing normal and PAM plus gRNAs into the CHO-K1 cells following the manufacturer's recommended protocols. To summarize the transfection process, 50 µL of DMEM/F12 (Gibco, USA) and plasmid DNA (1 µg) were combined in one tube. Simultaneously, 50 µL of DMEM/F12, along with lipofectamine® 3000 (1.5 µL) and 2 µg of p3000 reagent, were combined in another tube. After 5 minutes at room temperature, the two tubes were mixed and incubated for an additional 15 minutes at ambient temperature. Next, a gradual addition of the resultant mixture was made to the culture media's surface. The cells were kept in a 37°C incubator for 6 hours, in accordance with the specified conditions mentioned before. Afterward, a new DMEM/F-12 containing FBS was used in instead of the prior medium. 

**Isolation of single-cell cloning: **Using BD FACSAriaTM III fluorescence-based cell sorting (BD Biosciences), GFP-positive cell colonies were separated and plated in 96-well plates. Then, by cultivating the single cell clones in selected growth medium supplemented with an increased proportion of FBS, they were multiplied and grown. After a few days, the single cells underwent multiplication and increased in number. Once each clone had grown for a week, it was transferred to a 24-well plate, and DNA extraction was carried out from the proliferating clones.

***CASP8AP2*** **gene knockout confirmation by PCR amplification and sequencing: **Following the cell suspension, which contained around 1 × 10^6^ cells, was centrifuged, genomic DNA extraction was performed using the DENAzist Asia (Iran) DNA isolation and extraction kit, following the step-by-step instructions provided in the manufacturer's protocol. PCR test was performed to validate the deletion of the *CASP8AP2* gene and confirm the successful insertion of the PX460-1 vector at the target genomic location, using appropriate genomic primer sets. The donor vector's integrity was confirmed at the *CASP8AP2* site using two genomic primers and vector primers. Genomic primers were used to detect the presence of the PX460-1 vector, and a lack of PCR band indicated successful knockout.The PCR amplification was performed using a total 20 μL reaction mixture and then analyzed on a 1% agarose gel. For further confirmation, resulted PCR band was extracted from agarose gel and send for sequencing.

**Cell proliferation assay using MTT: **Both the CHO-K1 cells and CHO-*CASP8AP2* deficient (CHO-KO) cells were transferred to plates with 96 wells of cell culture at a density of 1×10^4^ cells per well and kept for duration of 24 to 120 hours at 37°C. Following the designated incubation period, each well was treated with a final concentration of 0.5 mg/ml of MTT reagent from sigma brand (USA). The plate was then placed in a CO_2_ incubator for 4 hours. After the 4-hour MTT treatment, the media containing MTT was removed entirely. In order to dissolve the generated formazan crystals by the viable cells, 100 μL of dimethyl sulfoxide (DMSO, Sigma Aldrich, USA) was added to all wells, and the plate was kept in the dark place for 15 minutes. Afterward, using an ELISA reader, the absorbance of each well was measured at 570 nm and 630 nm as reference wavelengths. Finally, data were statistically analyzed.

**Cell viability assay** **: **To evaluate cell viability, sodium butyrate (NaBu) was employed. Parental CHO-K1 cells and CHO-KO clones were added to each well of the 96-well plates at a density of 1 × 10^4^ cells per well. After 24 hours, the cells were exposed to different doses of NaBu (0 to 100 mM) and incubated for 24, 48 and 72 hours. At the respective time, MTT solution was added to the wells then in the incubator kept for four hours. Subsequently, each well received 100 μL of DMSO, and the plates were kept in the dark for 15 minutes. Utilizing an ELISA reader, absorbances were measured at reference wavelengths of 570 nm for each well. By contrasting it with the CHO-K1 control, the inhibitory concentration 50 (IC_50_) of CHO-KO was found. On the data, statistical analysis was done. To make sure that the results were accurate and consistency, this experiment was conducted three times with six duplicates for each iteration.

**Cell apoptosis assay:** Apoptosis assessment was performed using the Annexin V/PE and 7-AAD dyes from the BioLegend brand, which were included in the Apoptosis Detection Kit (USA). Around 1.5 × 10^5^ cells were vested in a 6-well plate then after 24 hours cells were exposed to a treatment of NaBu at a concentration of 3.5 mM. Subsequently, Trypsin was used to separate the cells, and they were then centrifuged. The cells were twice washed with a specific buffer and subsequently suspended in 100 μL of binding buffer. In the flowcytometry tube, a total of 5 µL of PE Annexin V and 5 µL of 7-AAD were added and gently mixed. After 15 minutes of incubation, binding buffer (400 µL) was added to the tube for 45 seconds before doing flow cytometric analysis. Three repetitions of this test were conducted.

**Cell cycle analysis using flow cytometry: **Two types of cells were planted in 24-well plates: CHO-K1 native cells and CHO-*CASP8AP2* defective cells. Each well contained 4 × 10^4 ^cells. To achieve a high confluence percentage, the cells were kept in the wells for 24 and 48 hours under optimal growth conditions. The cells were washed one or two times with PBS, then it was mixed with 4.5 ml of cold (70%) ethanol. After centrifugation, the suspended cells were incubated in 500 microliters of PBS containing 0.25% Triton X-100 at 4 degrees Celsius for duration of 15 minutes. The DNA was stained with 20 g/mL of propidium azide (PI), and RNase A and DNase-free (40 mg/mL) was also added. The cell suspension was again suspended in an additional 500 mL of PBS. Subsequently, they were incubated in a dark place for 30 minutes. A total of 10,000 cells were enumerated using flow cytometry for the purpose of cell cycle analysis. The data were examined using FlowJo software. FACS flow cytometry was utilized to determine the DNA content of the cell population. The data were examined using FlowJo software. 

**Statistical analysis: **SPSS Software (version 9.19; Chicago, IL, USA) was used to analyze data. One-way ANOVA, along with post hoc LSD and Tukey tests, were utilized for the statistical analysis of the performed tests. GraphPad Prism (version 8.0) was used to evaluate MTT tests and cell proliferation. For experiments involving three or more groups with two variables, two-way ANOVA was employed. Data from apoptosis and cell cycle assays were analyzed using FlowJo software (version 7.0). For all tests, a p-value less than 0.05 (*p*<0.05) were determined to be statistically significant. 

## Results

Two gRNAs were generated to selectively bind and facilitate the silencing of the *CASP8AP2* gene within its 3'UTR. Additionally, the "CRISPR-HITI" strategy was employed to specifically target the poly(A) signal sequence for efficient gene silencing. This strategy involved deleting a non-coding fragment that contained necessary elements for poly(A) tail formation, using two gRNAs, and simultaneously inserting a reporter gene.

PCR amplification followed by agarose gel electrophoresis was employed as a method to confirm the successful cloning of gRNAs in both PX459 and PX460-1 vectors. The amplification of standard gRNAs within PX459 yielded a discernible 220 bp product (Fig. S2-A). Concurrently, the cloning of gRNAs incorporating the PAM sequence in PX460-1 resulted in PCR products of 300 bp (Fig. S2-B). By following this experimental procedure, the cloning, and integration of the required gRNA constructs to the precise target location were effectively verified by confirming their presence in the respective vectors.

**Figure 1:**
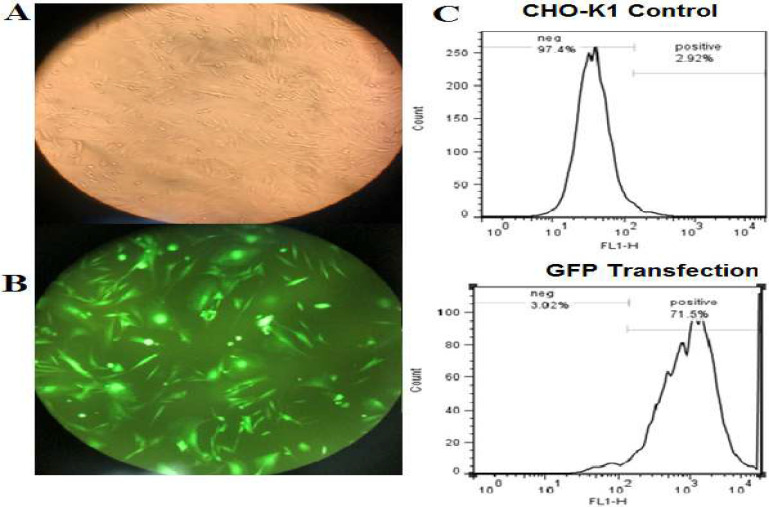
CHO-K1 cells transfected with CRISPR and donor vectors. A) Visible light microscopy. B) Fluorescent light microscopy. C) Flow cytometry analysis (71.5% transfection efficiency).

The two vectors, PX459 and PX460-1, were concurrently transfected into CHO cells using Lipofectamine®3000. These vectors contained normal gRNAs and gRNAs+ PAM, specifically targeting specific regions within the *CASP8AP2* gene. The number of transfected cells was counted using an inverted microscope equipped with fluorescent light. The results of this study indicated that approximately 70% of the cells displayed green fluorescent protein (GFP) when observed under the microscope, thereby indicating the effectiveness of the transfection process that had taken place (Fig. 1A, B). Flow cytometry was employed to accurately quantify the transfection efficiency, which showed a successful transfection rate of 71.5% (Fig. 1C).

**Figure 2:**
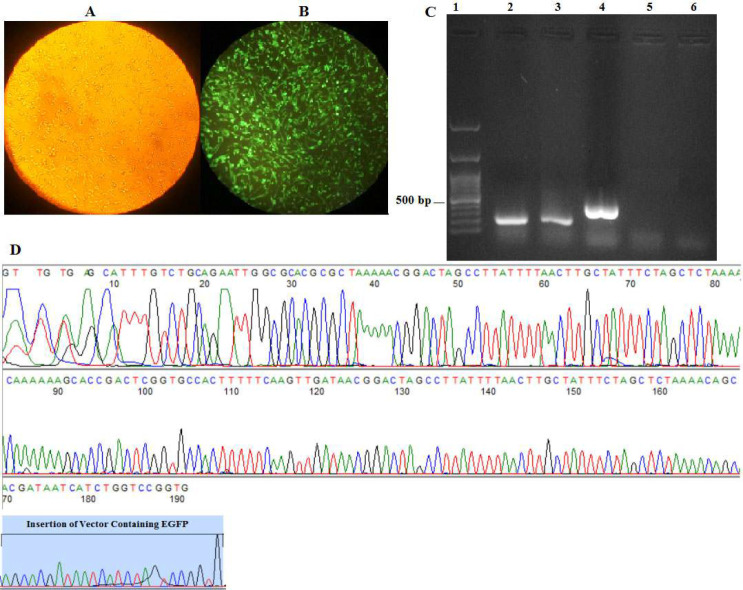
Clonal Selection and Verification of *CASP8AP2* Knockout in CHO Cells. A) CHO cells expressing EGFP under brightfield illumination (visible light) of an inverted microscope. B) Persistent EGFP expression in CHO cells visualized under the fluorescent light of an inverted microscope. C) Gel electrophoresis confirms knockout clone homozygosity (lanes 2 and 3) and EGFP integration (lane 4). Lane 1: ladder. Lane 5: No band due to large vector size. Lane 6: negative control. D) Sequencing confirms EGFP integration in *CASP8AP2*.

After 72 hours had elapsed from the successful transfection, single cells were isolated using cell sorting, following this, an EGFP-expressing cell colony expanded (Fig. 2A, B). PCR analysis was conducted on genomic DNA to validate the accurate insertion site of the knock-in. This PCR analysis showed that the EGFP cassette was correctly inserted into the *CASP8AP2* gene. Based on the length of the integrated DNA fragment exceeding 5000 bp, which exceeded the amplification capacity of conventional Taq polymerase, it was deduced that in homozygous clones where both alleles were deleted, the PCR test did not exhibit any bands, confirming homozygosity. In order to strengthen the confirmation process, additional primers were utilized as controls in the PCR analysis. These control primers yielded a 400 bp band, serving as validation of the PCR technique. Additionally, to confirm the deletion of the *CASP8AP2 *gene and the simultaneous insertion of the plasmid fragment containing GFP, forward and reverse cloning genomic primers were used. These primers amplified a PCR product with a fragment length of 270 bp and provided evidence for targeted genetic changes (Fig. 4C). Finally, the successful deletion of *CASP8AP2* gene in CHO cells in the 3ʹUTR region was confirmed by sequence analysis (Fig. 2D).

**Figure 3:**
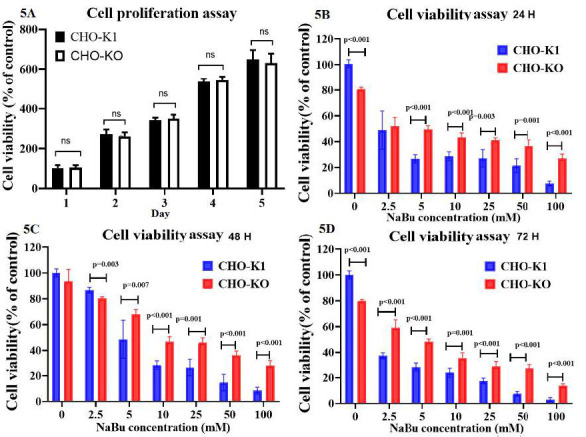
Cell proliferation assays and Cell viability of CHO-K1 cells following exposure to sodium butyrate (NaBu) by using MTT. A) Cell proliferation assays. B-D) Cell viability after exposure to NaBu for 24, 48, and 72 hours, respectively. CHO-K1 cells exhibited higher viability than CHO-*CASP8AP2* knockout cells across all time points and NaBu concentrations.

To examine the effect of deleting the *CASP8AP2* gene on cell proliferation rate, clones of CHO-K1 cells with the deleted *CASP8AP2* gene and a control group were cultured in a suitable medium for the initial five days. The MTT technique was used to determine the cell proliferation rate. The results indicated that the deletion of the *CASP8AP2* gene did not affect the CHO-KO clone's cell proliferation ability compared to the control group (CHO-K1) at any of the examined time points. As a result, there was no significant difference observed in the cell proliferation ability between the two groups (*p* > 0.05) (Fig. 3A).

 To evaluate the potential influence of *CASP8AP2* knockout on cell viability, an MTT assay was conducted at 24, 48 and 72 hours using different concentrations of NaBu (0, 2.5, 5, 10, 25, 50, and 100 mM). This study demonstrated that the silencing of *CASP8AP2* gene increased cell viability in CHO-KO cells as opposed to CHO-K1 cells at 24, 48 and 72 hours. Through statistical evaluation with the ANOVA test, the discrepancies were verified (Fig. 3B-3D). Specifically, the CHO-KO cells with *CASP8AP2* gene knockout exhibited a higher IC_50_ value than the control group (CHO-K1) at these time points. The IC_50_ values for knockout cells and native cells were determined to be 7.84 mM and 3.43 mM, respectively. At 48 hours, the IC_50_ values were measured as 15.82 mM for CHO-KO and 6.43 mM for CHO-K1. At 72 hours, the IC_50_ values were measured as 5.69 mM for CHO-KO and 2.02 mM for CHO-K1. These findings suggest that disrupting the *CASP8AP2* gene, which plays a pro-apoptotic role, contributes to the enhanced viability of CHO-KO cells. The results from the cell viability assay further revealed a significant increase in cell viability after 24 hours. 

**Figure 4:**
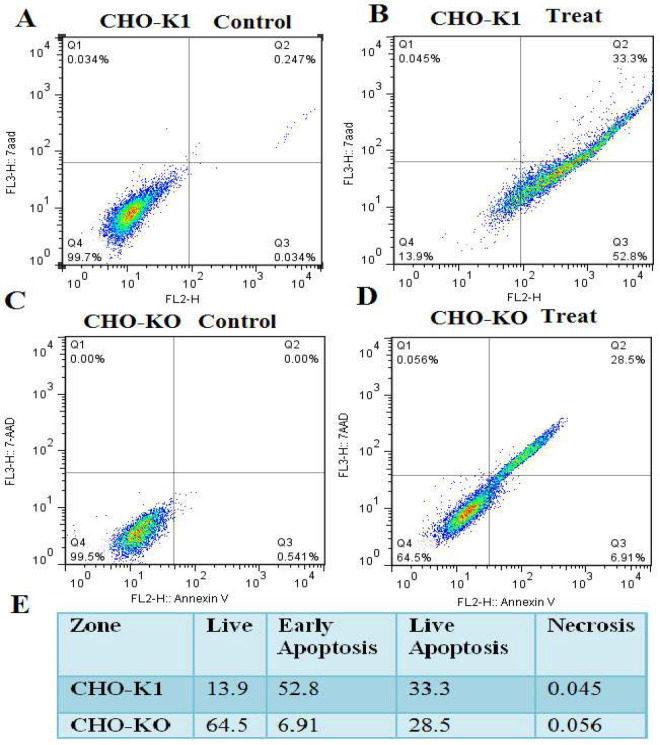
Apoptosis assay in CHO-K1 and *CASP8AP2*-KO cells. A-D) Histograms for control and NaBu-treated (3.5mM) CHO-K1 (A, B) and *CASP8AP2* KO (C, D) cells. E) Quantification of apoptotic cell percentages.

To evaluate the apoptosis status of *CASP8AP2* knockout cells, flow cytometry analysis was performed. Both the CHO-K1 cell line (control group) and CHO-KO (CHO-*CASP8AP2* silencing) were subjected to NaBu exposure at a concentration of 3.5 mM. Additionally, the research comprised blank control groups made up of CHO-K1 and CHO-KO cells that had not received any NaBu treatment. The evaluation was repeated three times over 24 hours. The results revealed that 86.1% of CHO-K1 cells underwent apoptosis, with 52.8% in the early apoptosis phase and 33.3% in the late apoptosis phase. Only 13.9% of CHO-K1 cells remained viable. In contrast, the CHO-KO displayed greater resistance than wild-type cells. Among the knockout clone, 6.91% of cells were in the early apoptosis phase, with only 28.5% in the late apoptosis phase, and 64.5% remaining alive (Fig. 4). 

**Figure 5:**
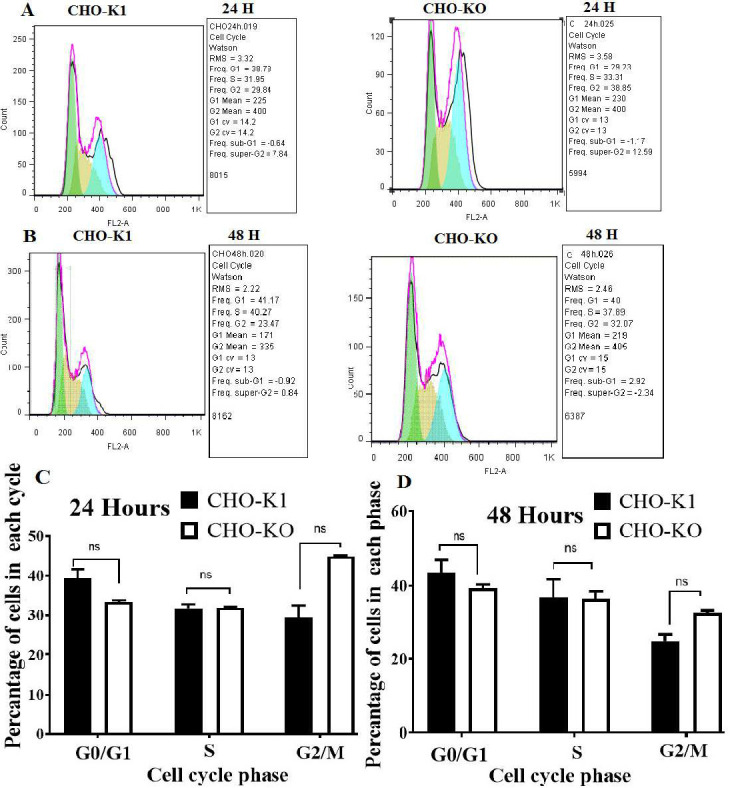
Flow Cytometry Analysis of Cell Cycle Progression in CHO-K1 and CHO-KO Cells. A) Propidium iodide staining reveals no difference in cell cycle distribution at 24 hours. B) After 48 hours, CHO-K1 cells have more G2/M phase cells and fewer S phase cells than CHO-KO cells (*p *< 0.05). C and D reveal the graph on cell cycle phases in both times. ns = not significant.

The deletion of the *CASP8AP2* gene, an essential regulator of cell cycle progression, resulted in notable changes to the cell cycle progression of CHO-K1 cells. However, these changes were not statistically significant. Compared to native cells, CHO-KO cells had a smaller percentage of G1 phase cells, and the rate of cell proliferation in the S phase and the G2 phase was higher in CHO-KO cells than in native cells. The fraction of accumulated G1 phase cells was reduced in CHO-KO cells, and in CHO-KO cells, the deletion of the *CASP8AP2* gene resulted in a higher proportion of living cells accumulating in the G2 phase (Fig. 5).

## Discussion

To enhance protein production in CHO cells, we used CRISPR/Cas9 to silence the apoptosis regulator *CASP8AP2*, aiming to reduce apoptosis and increase cell viability. The exact mechanism is unclear, but it may involve disrupting interactions with apoptosis-related proteins or hindering caspase-8 activation, and the 3’UTR region of the *CASP8AP2* gene may also play a role in regulating its expression [14]. 

The *CASP8AP2* gene was effectively knocked out by targeting its 3’UTR with a CRISPR-HITI strategy, a process confirmed by PCR, sequencing, and corroborated by Kumar and colleagues. The integration of an EGFP expression cassette into the poly(A) signal sequence suppressed *CASP8AP2* expression, demonstrating the efficiency, ease, and specificity of CRISPR for 3’UTR knockout and its utility in studying gene regulation [15, 16]. 

Two types of guide RNA (gRNA) were designed for efficient *CASP8AP2* targeting and its 3’UTR regulation. Despite the challenges of 3’UTR targeting and PAM sequence restrictions, scientists have explored gRNA-free one-step genome editing with CRISPR/Cas9 for precise alterations [17].

Studies have shown that deletion of *CASP8AP2* can have varying effects on cell proliferation in different cell types. The MTT assay revealed that CHO-KO clones with deleted *CASP8AP2* showed a time-dependent increase in proliferation rates. However, this increase was not statistically significant compared to the control group. The results align with the conclusions of earlier research that showed *CASP8AP2* knockout cells did not exhibit any appreciable changes in cell proliferation [18, 19]. 

Another aspect of this study was to examine the impact of deletion of the *CASP8AP2* gene on the viability of CHO-KO cells compared with the control group. *CASP8AP2* gene encodes a protein called FLASH, which is component of a variety of cellular processes, including cell death, DNA repair, and cell cycle regulation. Research has demonstrated that FLASH is necessary for both healthy and malignant cells to survive. 

Sodium butyrate, the sodium salt of butyric acid, was used to study its effects on the survival of *CASP8AP2* gene-deleted CHO cells, with its ability to modulate gene expression, inhibit cell growth, and trigger apoptosis [20]. This compound's impact on cell proliferation, apoptosis induction, and cell cycle regulation was the focus of our study, given its proven efficacy in halting the cell cycle and reducing the growth rate of CHO cells. Notably, Avello et al. (2022) reported a decrease in the growth rate of CHO cells cultured in the presence of sodium butyrate, a finding that aligns with the results obtained in our study [21].

Researchers have investigated the impact of sodium butyrate on various cell lines beyond CHO cells. Studies have revealed that butyrate exerts anti-proliferative and pro-apoptotic effects on HCT116 colorectal cancer cells [22]. We showed that *CASP8AP2* played a crucial role in regulating apoptosis, particularly in response to sodium butyrate treatment. Furthermore, an investigation into the influence of sodium butyrate on IPEC-J2 cells demonstrated its capacity to suppress cell proliferation through two mechanisms: induction of cell cycle arrest at the G0/G1 phase and promotion of apoptosis at elevated concentrations [23]. Our study revealed that deletion of the *CASP8AP2* gene improved the viability of engineered cells compared to normal CHO-K1 cells. This resistance was observed even at 100 mM sodium butyrate, a compound known to induce apoptosis. These findings suggest that suppressing *CASP8AP2* can increase the resistance of cells to sodium butyrate-induced apoptosis. It is worth noting that the concentration range (0-100 mM) of sodium butyrate was selected based on a study conducted by C McCabe in 2019 [24].

Another crucial objective of this research was to evaluate the effects of *CASP8AP2* gene deletion on apoptosis in CHO knockout cells. The results of this study also showed that the clones in which the *CASP8AP2* gene was deleted were more resistant to apoptosis than CHO-K1 cells when exposed to the drug sodium butyrate. These findings of resistance to apoptosis were consistent with our results from the cell viability test and confirmed the importance of *CASP8AP2* in apoptosis process. The proposed mechanism by which *CASP8AP2* exerts its anti-apoptotic effect is through modulation of caspase activity and upregulation of the anti-apoptotic gene *XIAP*. This suggests that *CASP8AP2* acts as a gatekeeper, preventing uncontrolled activation of caspases, which are essential for triggering apoptosis [19]. Furthermore, it has also been determined that the *CASP8AP2* gene is directly or indirectly involved in signaling pathways related to the regulation of cell proliferation and death. These pathways may include PI3K/Akt or MAPK signaling pathways and their dependent genes, such as *MAP3K7f*, which are effective in regulating nucleic acid production and cellular activities [25]. Our results align with earlier research in this field. A salient example is the study conducted by Kling et al. (2020), which demonstrated that the deletion of *CASP8AP2* in the 3'UTR region by miR-210 resulted in a reduction in apoptosis [26].

Our research on the deletion of the *CASP8AP2* gene in CHO cells revealed a growth slowdown or halt in the G2/M phase, with a 10% rise in cell count in this phase after 24 hours, aligning with previous studies on mouse embryonic stem cells. These findings not only enhance our understanding of cell death and survival mechanisms but also suggest the potential for manipulating *CASP8AP2* expression as a novel therapeutic approach for diseases marked by abnormal cell death or survival [18]. 

In contrast, a comparable study on HEK cells revealed that deletion of *CASP8AP2* led to G0/G1 phase arrest, resulting in a 7-fold rise in protein synthesis. This difference could be due to the different cell types from the two different species of origin [19].

Despite the fact that the gene did not influence cell proliferation in a substantial way and the overall cell cycle, In fact, the suggestion that *CASP8AP2* functions as a regulatory gene implies that it has a role in controlling the expression of other genes. The significance of further investigation to clarify the exact mechanisms by which *CASP8AP2* affects gene expression is highlighted by its regulatory function. These processes might entail interactions between proteins or the synthesis of non-coding RNA. A better understanding of how *CASP8AP2* functions as a regulatory gene could lead to strategies for enhancing recombinant protein yields in CHO cell-based bioproduction systems.
