# Supplementary material for: A CRISPR-HITI strategy approach to improve CHO cell viability by modifying the 3'UTR of Caspase 8 Associated Protein 2
Source: Mol Biol Res Commun. 2025;14(1):15–26. doi: 10.22099/mbrc.2024.50513.2000 (PMC11624615; doi:10.22099/mbrc.2024.50513.2000)
Supplement: Supplementary file 1 — Figure S1, S2 [file mbrc-14-15-s001.pdf]

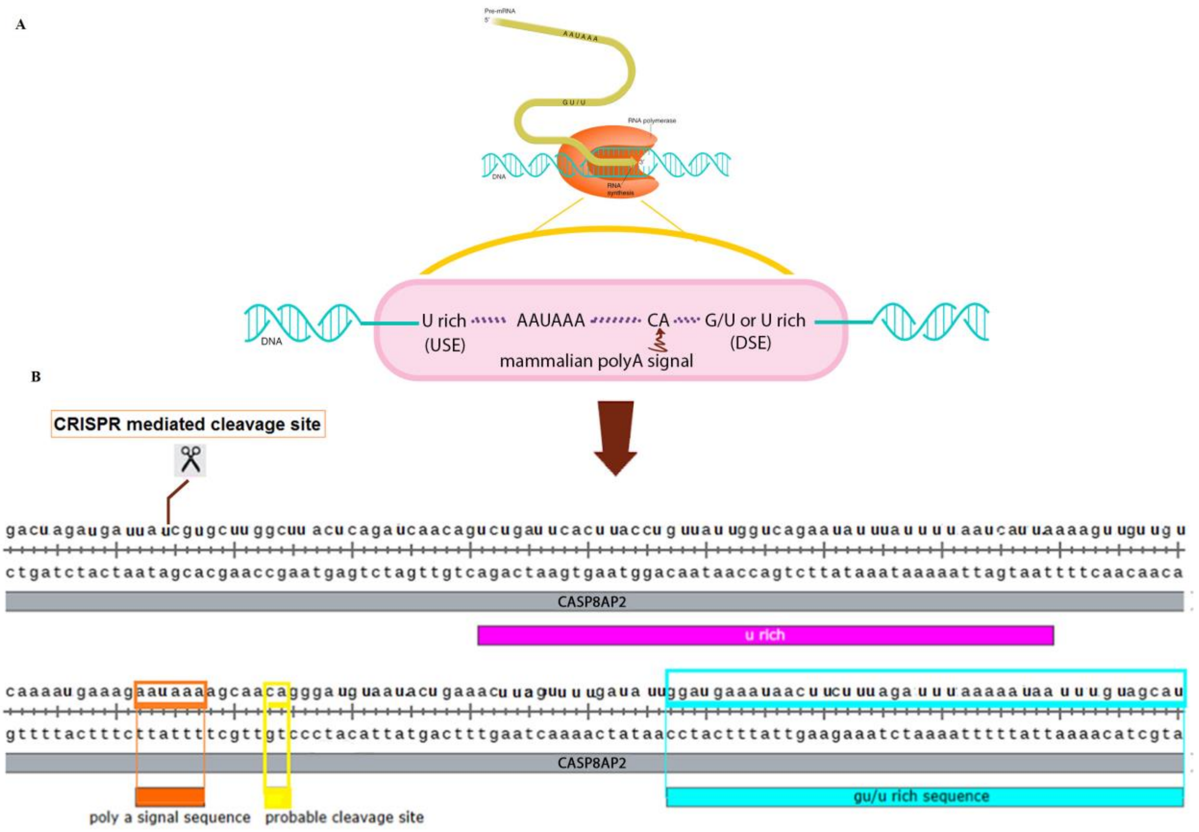

Figure S1: Targeting strategy using CRISPR system on the 3'UTR of *CASP8AP2* gene.

A) Schematic representation of the 3'UTR context and structure. B) Design of gRNAs for CRISPR-Cas9 mediated cleavage. The gRNAs target the 3'UTR of *CASP8AP2* gene, resulting in the removal of a non-coding fragment containing the poly(A) signal sequence. The HITI strategy is then used to insert an EGFP reporter cassette at the cleavage site.

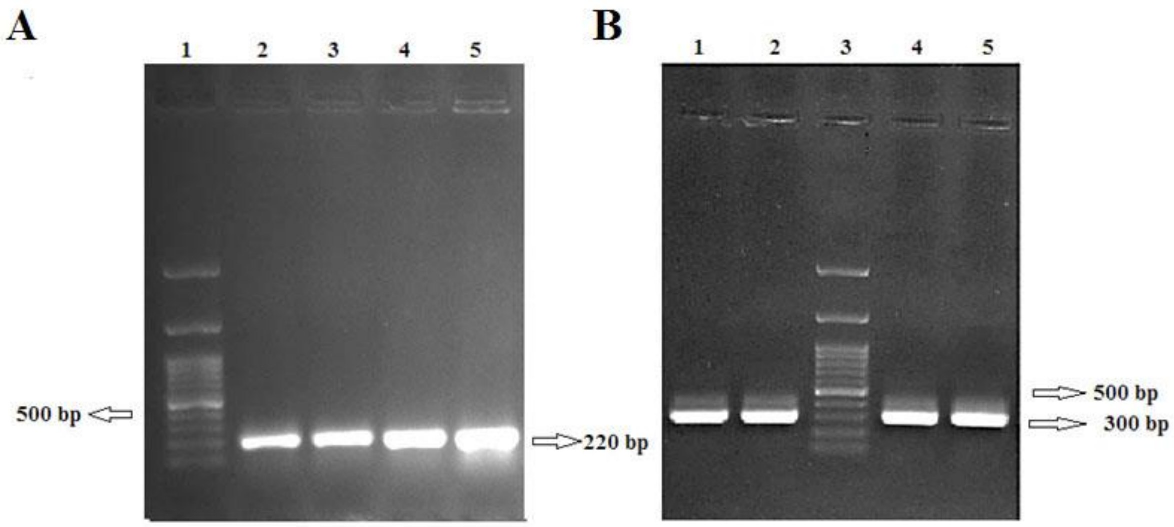

Figure S2: PCR validation of gRNA cloning in PX459 (220 bp) and PX460-1 (300 bp) plasmids.

A) PX459, B) PX460-1. Lanes: 1: 100 bp ladder, 2, 4: gRNAs, 3, 5: gRNAs with PAM.
