# Supplementary material for: A CRISPR-HITI strategy approach to improve CHO cell viability by modifying the 3'UTR of Caspase 8 Associated Protein 2
Source: Mol Biol Res Commun. 2025;14(1):15–26. doi: 10.22099/mbrc.2024.50513.2000 (PMC11624615; doi:10.22099/mbrc.2024.50513.2000)
Supplement: Supplementary file 2 — Table S1 [file mbrc-14-15-s002.pdf]

Table S1: Primers and sequences used for PCR

Name	Sequences
**Genomic primers**	Fw:5′ GGATTTTACGGCGTTTCATTTA3′<br>Rev: 5′TCCCTGTTGCTTTTATTCTTTCA3′
**Cloning primer**	Fw: 5′ GCCTTTTGCTGGCCTTTTGCTC3′<br>Rev: 5′CGGGCCATTTACGTAAGTTATGTAACG3′
**Positive control**	Fw: 5′CACCgAGATGGCGTGACGCCAATAA3′<br>Rev: 5′AAACCGGCATGCCTAAAGCGTATC3′
